# Case report: SCN4A p.R1135H gene variant in combination with thyrotoxicosis causing hypokalemic periodic paralysis

**DOI:** 10.3389/fneur.2022.1078784

**Published:** 2023-01-17

**Authors:** Zhi Zhang, Banghui Xiao

**Affiliations:** Department of Endocrinology and Metabolism, Affiliated Hospital of Guizhou Medical University, Guiyang, China

**Keywords:** hypokalemic periodic paralysis, SCN4A gene, thyrotoxic periodic paralysis, hyperthyroidism, therapy

## Abstract

Hypokalemic periodic paralysis (HPP) is a heterogeneous group of diseases characterized by intermittent episodes of delayed paralysis of skeletal muscle with episodes of hypokalemia, caused by variants in CACNA1S or SCN4A genes, or secondary to thyrotoxicosis, Sjogren syndrome, primary aldosteronism, etc. HPP may be the only presentation in Andersen–Tawil syndrome in which the majority of cases are caused by pathogenic variants in the KCNJ2 gene. We present a case of a 29-year-old male with hypokalemic periodic paralysis. The patient began to experience recurrent weakness of the extremities at the age of 26, which was effectively treated with potassium supplementation. He had recently developed dry mouth, palpitations, weight loss, and even dyspnea, with a serum potassium level as low as 1.59 mmol/L. The results of auxiliary examinations showed Graves' disease, and genetic testing indicated a missense variant, NM_000334.4 (SCN4A):c.3404G>A (p.R1135H). He did not experience periodic paralysis during follow-up after lifestyle guidance and treatment of thyrotoxicosis with radioactive iodine. It is a rare case of SCN4A p.R1135H gene variant combined with hyperthyroidism resulting in HPP with respiratory muscle paralysis to raise awareness of the disease and avoid misdiagnosis and missed diagnosis.

## Introduction

Hypokalemic periodic paralysis (HPP) is a rare ion channel disorder and is one of the most common forms of periodic paralysis. The incidence of HPP is ~1 in 100,000 (1). Since Musgrave proposed HPP in 1,727 (2), there has been a growing understanding of the disease. HPP is caused primarily by variants in genes encoding skeletal muscle ion channels that reduce the excitability of the muscle membrane, leading to susceptibility to episodes of paralysis (3). The disease is genetically heterogeneous, and the known variants are mainly in the CACNA1S gene, which encodes a voltage-gated calcium channel in skeletal muscle, and the SCN4A gene, which encodes a sodium channel, among which the CACNA1S gene variant predominates, accounting for about 60% of all variants and the SCN4A gene variant for 20% (4). Approximately 20% of HPP remain unidentified genetically. The disease is generally autosomal dominant and is more common in non-Hispanic Caucasians, known as familial HPP (FPP) (5). Non-familial HPP includes thyrotoxic periodic paralysis (TPP) and sporadic periodic paralysis (SPP), which are more common in Asians and Hispanics (5). The incidence of SPP in Asian populations is lower than that of TPP which is about 2% in Asian populations with thyrotoxicosis (6). Graves' disease (GD) is the most common cause of TPP (7). The pathogenesis of sporadic periodic paralysis is unclear. Sung et al. conducted a study to clarify whether sporadic HPP and familial HPP have similar genotypes and showed that all FPPs contained variants in the CACNA1S or SCN4A genes, whereas only 4 of 60 participants with SPP were detected with variants in the CACNA1S or SCN4A genes, and there was no significant difference in phenotype between SPP and FPP with variants, except for the later age of onset of SPP (8). The mechanism of hyperthyroidism leading to HPP is also not clear. TPP is associated with elevated thyroid hormone-stimulated Na^+^-K^+^ ATPase activity. TPP was suggested to be an ion channelopathy, and variants in KCNJ18 encoding potassium channel Kir2.6 were reported in some TPP patients (9–11). Interestingly, variants in the KCNJ18 gene were also reported in patients with SPP (10). There are now substantial studies showing that variants in SCN4A or CACNA1S gene are not associated with TPP (12). The combination of coexisting HPP caused by SCN4A gene variants and thyrotoxic periodic paralysis is very rare. We present an exceptionally rare case of the coexistence of HPP caused by variants in SCN4A genes and TPP.

## Case description

A 29-year-old Chinese male patient, who started to experience recurrent weakness in the morning or after staying up late at the age of 26, was diagnosed with HPP associated with a genetic variant after a complete examination at a local hospital, but the specific gene was not clear. We were unable to retrieve detailed data on relevant laboratory parameters at that time, but he remembered clearly the thyroid function was normal. Treatment with potassium supplementation was effective, but recurrent episodes persisted. He was admitted to the local hospital 12 days before he came to our hospital due to an increased frequency of attacks, muscle pain, dry mouth, palpitation, and profuse sweating. The serum potassium level was 1.59 mmol/L. Intravenous potassium supplementation was not effective, and he had chest tightness, inability to speak, and dyspnea. Blood gases suggested PH 7.18, PCO_2_ 54.8 mmHg, and PO_2_ 134 mmHg. After endotracheal intubation, mechanical ventilation, and potassium supplementation, the above symptoms improved, but they still recurred and his weight dropped by 2 kg. He denied any past medical history and taking other medications. His parents were healthy and he was an only child, and there were no similar symptoms in his family.

Physical examination showed that vital signs all remain stable and in a normal range. The heart was in rhythm, no significant murmurs were heard in all valve auscultation areas, and his muscle strength and tone were normal.

Laboratory examination revealed thyroid-stimulating hormone (TSH) of 0.01 mIU/L (reference interval, RI:0.27–4.20), free thyroxine (FT4) of 28.42 pmol/L (RI:12.00–22.00), free triiodothyronine (FT3) of 9.98 pmol/L (RI:3.10–6.80), thyrotropin receptor antibody (TRAb) of 20.82 IU/L (RI:0.00–1.75), serum potassium of 2.93 mmol/L (RI:3.5–5.3), sodium of 142.35 mmol/L (RI:137–147), chloride of 110.41 mmol/L (96–108), calcium of 2.09 mmol/L (RI:2.11–2.52), magnesium of 0.69 mmol/L (RI:0.75–1.02), inorganic phosphate of 1.19 mmol/L (RI:0.85–1.51), and carbon dioxide combining power of 20.32 mmol/L (RI:22–29). Hepatic and renal function, serum muscle enzymes, blood glucose, cortisol, renin, and aldosterone were within normal limits. Antinuclear antibody, SSA, SSB, anti-thyroglobulin antibody, and antithyroid peroxidase antibody were negative. Electrocardiogram showed sinus rhythm and marked T depression in leads I, aVL, II, III, aVF and V5–V6, and abnormal U-wave. Static thyroid imaging showed the fullness of the thyroid gland bilaterally with diffuse hyperintensity, consistent with “hyperthyroidism.” The thyroid ultrasound did not show any significant abnormalities. The iodine uptake rate of the thyroid gland was higher than normal. Genetic testing revealed a pathogenic heterozygous variant, NM_000334.4 (SCN4A):c.3404G>A (p.R1135H) after obtaining the patient's informed consent. Regretfully, due to distance, the patient's parents failed to undergo genetic testing.

Based on these results, we diagnosed the patient with hypokalemic periodic paralysis, caused by a variant in the SCN4A gene combined with Graves' disease. Lifestyle education was given, and treatment with radioactive iodine was administered. The patient was followed up at 3 and 9 months after discharge. He stated no recurrence of muscle pain and weakness after discharge and became hypothyroid (Table 1), and he is now being given 100 μg of levothyroxine replacement therapy.

**Table 1:** Thyroid function before treatment and during follow-up.

**Variable**	**Before treatment**	**3 months at follow-up**	**9 months at follow-up**
TSH (mIU/L)	0.01	236.40	66.34
FT4 (pmol/L)	28.42	<0.30	2.17
FT3 (pmol/L)	9.98	17.70	5.11

## Discussion

Hypokalemic periodic paralysis associated with variants in the CACNA1S or SCN4A genes, also known as primary HPP and thyrotoxic hypokalemic periodic paralysis, did not differ in terms of attack characteristics, with prodromal symptoms including muscle pain, fatigue, and paresthesia. Weakness usually begins in the proximal muscles of the lower extremities and may progress to flaccid tetraplegia. Episodes of HPP have certain triggers, including ingestion of carbohydrates or alcohol intake, and rest after strenuous exercise. The paralytic attack rate of primary HPP is highest between the ages of 15 and 35 years and decreases after the age of 35 years. The muscle weakness can be focal or generalized, with the ocular, bulbar, and respiratory muscles usually unaffected, lasting several hours (sometimes up to several days) and then gradually subsiding (1, 2). TPP usually occurs in people around 20–40 years of age, with gender differences, with a male-to-female ratio of about 20:1 (12). A case accompanied by ventricular tachycardia and acute respiratory failure has been reported (13), but life-threatening complications have rarely been seen in clinical practice.

In this case, the patient had typical clinical features of HPP. He was a young Asian man, with typical muscle aches and pains during attacks, triggered by staying up late, and potassium supplementation was effective. The patient was initially diagnosed with HPP associated with a gene variant at a local medical unit. As the patient grew older, the frequency of episodes did not decrease, and he developed symptoms of hyperthyroidism such as dry mouth, palpitation, and weight loss. In addition, potassium can even be as low as 1.59 mmol/L, and respiratory muscles were involved, leading to respiratory muscle paralysis.

The diagnosis of primary HPP is based on a history of flaccid paralysis episodes, a positive family history, characteristic changes in serum potassium, and the exclusion of secondary causes. Whereas, genetic testing is the gold standard for confirming the diagnosis, a significant number of patients have no identifiable variants (3). Diagnostic clues for TPP include adult male, no familial hypokalemic periodic paralysis, no familial history of hyperthyroidism, or hypophosphatemia at the time of the episode, blood acid–base balance is relatively normal (12), and thyrotoxicosis is a prerequisite for the diagnosis of TPP. For our patient, the possibility of HPP due to Sjogren syndrome, Cushing syndrome, and primary aldosteronism was excluded. There were no hyperthyroid symptoms, the thyroid function was normal at that time of onset, and he had no clues at the time to suggest secondary periodic paralysis.

The main sodium channel expressed in skeletal muscles is Nav1.4, which alpha subunit is encoded by SCN4A (4). The alpha subunit of the sodium channel consists of four homologous domains, designated DI to DIV. Each domain is composed of six transmembrane helical segments named S1 to S6, and segments S1–4 form the voltage-sensing domain (14). Since Bulman et al. found that the variant of R669H of SCN4A was associated with the pathogenesis of HPP in 1999 (15), 10 SCN4A variants have been linked to HPP, and HPP caused by variants in SCN4A genes is inherited with an autosomal dominant trait (4). Functional studies suggest that the most likely pathomechanism consists of the creation of an aberrant pore beside the normal ion conduction pathway. This gating pore permits small leakage of cationic currents at rest, favoring membrane depolarization in hypokalemia that, in turn, inactivates sodium channels and rends the fiber inexcitable (4). The variant (c.3404G>A: p.R1135H) is in the S4 segment of domain III and causes HPP by neutralizing arginine residues in S4 segments (16). The variant was classified as variants of pathogenic according to ClinVar. Functional studies with native R1135H muscle fibers showed that the variant increases the probability of depolarized resting membrane potentials under normal conditions and reduced extracellular potassium compatible with the diagnosis (17). R1135H has been reported in heterozygous patients with FPP and also can be *de novo* variants in SPP patients (8, 16, 17). The prevalence study of genetically defined skeletal muscle channelopathies in England showed that the frequency of R1135H in 95 HPP patients was 4% (18). The individuals with the variants had typical HPP phenotype, and the age at onset of attacks was in the second decade, with attacks usually occurring at night or in the early morning, and associated with low serum potassium levels or with provocative factors (16).

Although the results of gene testing at the time of onset were not known, we still considered that the cause of the periodic paralysis initial episode due to a genetic variant associated with primary HPP (c.3404G>A: p.R1135H in SCN4A), exacerbated by hyperthyroidism even leading to dyspnea.

This reminds us that although we identified the cause of HPP through gene testing, we still need to pay attention to whether the patient has other causes leading to changes in the condition, and we need to dynamically monitor other indicators to prevent life-threatening situations.

Treatment of primary HPP should be multifaceted, beginning with lifestyle and behavioral changes, such as identification and avoidance of triggers and avoidance of high carbohydrate intake (3). Pharmacologic treatment for HPP currently focuses on potassium supplementation, potassium-preserving diuretics, and carbonic anhydrase inhibitors (including acetazolamide and dichlorphenamide). Of these, oral or intravenous potassium supplementation is the only way to abort the paralysis. Acetazolamide and dichlorphenamide are commonly used for the prevention of periodic paralytic episodes, but the mechanism of action remains unclear. Acetazolamide has been the most commonly used drug for the treatment of HPP since 1968. A retrospective study in the UK showed that only about 50% of patients with primary HPP responded to acetazolamide, and patients with the CACNA1S variant had a greater chance of benefit than patients with the SCN4A variant (19), while exacerbations have been reported with acetazolamide in patients with the SCN4A variant (20). Dichlorphenamide was the first drug in the US to be approved for use in patients with primary hypokalemic periodic paralysis, and several clinical trials have shown that dichlorphenamide is effective in reducing the frequency of episodes of primary HPP (21), and in improving the quality of life of patients with HPP and maintaining its efficacy over time, despite the relationship between genetic background and the effectiveness of dichlorphenamide remains unknown (22). However, common side effects of using carbonic anhydrase inhibitors include abnormal sensation, fatigue, mild cognitive impairment, and kidney stones (3). Potassium-preserving diuretics have also been effective in primary HPP in patients who are intolerant to carbonic anhydrase inhibitors. Some studies have suggested bumetanide as a potential treatment for periodic paralysis, but the sample size of the relevant studies is small, so the potential use of this drug for the treatment of HPP requires further study (23). Treatment of TPP includes potassium chloride supplementation, non-selective β-blockers to prevent episodes of TPP by inhibiting sodium–potassium ATPase activity, antithyroid drugs, radioactive iodine, surgical control of hyperthyroidism, and avoidance of precipitating factors. In contrast, acetazolamide, a classic drug for primary hypokalemic periodic paralysis, can induce recurrent episodes of TPP (12). Because the treatment methods are different, it is very important to identify the cause. A study showed that to minimize future relapses, more definitive primary treatment such as radioactive iodine or surgery is preferred over ATD alone (24). For this patient, as a young male patient with SCN4A gene variant combined with Graves' disease, the symptoms were severe at the time of the attack; hence, we ultimately recommended that the patient's symptoms improve with lifestyle interventions, treatment with radioactive iodine for thyrotoxicosis, and he did not experience attacks of periodic paralysis.

## Conclusion

We present an exceptionally rare case of hypokalemic paralysis caused by a variant, NM_000334.4 (SCN4A):c.3404G>A (p.R1135H), combined with hyperthyroidism. From this case, we conclude that even after the genetic diagnosis is clear that the variant causes hypokalemic periodic paralysis, it is still necessary to pay attention to dynamic monitoring of thyroid function and other causes of periodic paralysis in order to avoid life-threatening exacerbation of the patient's condition and to reduce misdiagnosis and underdiagnosis.

## Data availability statement

The original contributions presented in the study are included in the article/supplementary material, further inquiries can be directed to the corresponding author.

## Ethics statement

Ethical review and approval was not required for the study on human participants in accordance with the local legislation and institutional requirements. The patients/participants provided their written informed consent to participate in this study. Written informed consent was obtained from the individual(s) for the publication of any potentially identifiable images or data included in this article.

## Author contributions

ZZ and BX substantially contributed to the collection of the data, participated in drafting, and approved the final version for publication. All authors agree to be accountable for the content of the work. All authors contributed to the article and approved the submitted version.
